# COVID-19 and *Burkholderia cepacia* co-infection in pregnancy associated with fetal demise: a case report

**DOI:** 10.11604/pamj.2022.42.173.33813

**Published:** 2022-07-04

**Authors:** Brenda Abena Ampah, Nana Kwame Ayisi-Boateng, Augustina Angelina Sylverken, Japhet Senyo, Kennedy Gyau, Betty Nkansah Osei Mensah, Godfred Acheampong, Michael Owusu

**Affiliations:** 1 University Hospital, Kwame Nkrumah University of Science and Technology, Kumasi, Ghana,; 2 Department of Medicine, Kwame Nkrumah University of Science and Technology, Kumasi, Ghana,; 3 Department of Theoretical and Applied Biology, Kwame Nkrumah University of Science and Technology, Kumasi, Ghana,; 4 Department of Clinical Microbiology, Kwame Nkrumah University of Science and Technology, Kumasi, Ghana,; 5 Centre for Health System Strengthening, Kumasi, Ghana,; 6 Department of Medical Diagnostics, Kwame Nkrumah University of Science and Technology, Kumasi, Ghana

**Keywords:** COVID-19, *Burkholderia cepacia*, pregnancy, SARS-CoV-2, case report

## Abstract

Since the global pandemic of the 2019 coronavirus disease (COVID-19), few studies have reported on the relevance of bacteria co-infection on outcome of COVID-19 patients. Little is known about the clinical presentation among pregnant women, mother-to-child transmission, and fetal outcomes. This report shows a 24-year-old nulliparous woman who was 32 weeks pregnant and was admitted to the University Hospital, Kwame Nkrumah University of Science and Technology (KNUST), Kumasi Ghana with symptoms of fever (40.3°C), cough and breathlessness of two weeks duration. Her nasopharyngeal sample tested positive for Severe Acute Respiratory Syndrome Coronavirus-2 (SARS-CoV-2) and blood culture isolated Burkholderia cepacia. She was given medications but went into pre-term labour and delivered a stillborn baby. This rare case of COVID-19 and Burkholderia cepacia co-infection emphasizes the need for a thorough assessment and appropriate treatment of patients presenting with fever and respiratory symptoms in order to mitigate poor outcome.

## Introduction

The 2019 Coronavirus Disease (COVID-19) is an infectious disease caused by a novel RNA virus, Severe Acute Respiratory Syndrome Coronavirus-2 (SARS-CoV-2) [1]. The virus was first detected in Wuhan, China in December 2019 and has been associated with very high morbidity and mortality rates. It usually presents with upper respiratory symptoms such as fever, cough, sneezing, runny nose, headaches, and breathlessness, and in advanced cases can lead to COVID-19 pneumonia, acute respiratory distress, and ultimately death [2]. However, the majority of cases are either mild or asymptomatic, and this is more common in children and young adults [3]. Due to their low immunity, pregnant women infected with COVID-19 are more likely to be hospitalized and are at increased risk for Intensive Care Unit (ICU) admission and receipt of mechanical ventilation than non-pregnant women [4]. Although information is limited, few developing countries have reported on COVID-19 related maternal and fetal outcomes [5]. Studies on SARS and Middle East Respiratory Syndrome (MERS-CoV) coronaviruses emphasized the role of co-bacterial infection [6]. A case report of *B. cepacia* in COVID-19 indicated that the co-infection may complicate COVID-19 [7]. However, report on this is limited to sub-Saharan Africa. We therefore present a rare case of COVID-19 and *Burkholderia cepacia* co-infection in pregnancy with associated poor fetal outcome. We seek to highlight the clinical presentation, diagnostic investigations and case management in a resource-limited setting.

## Patient and observation

**Patient information:** a 24-year-old pregnant woman (nulliparous at 32 weeks) presented to the University Hospital, Kwame Nkrumah University of Science and Technology (KNUST) [8], Kumasi, Ghana with complaints of cough, fever, chills, and breathlessness of about 2 weeks duration. Prior to the onset of symptoms, she was a regular attendant at Antenatal Care (ANC), booked at 22 weeks+4 days, and had an uneventful pregnancy. Symptoms started 2 weeks prior to presentation with cough, fever, chills which later became progressive and associated with breathlessness after 1 week.

**Clinical findings:** on examination, the temperature was 40.3°C at first, but dropped back to normal (37.6°C) after 5 hours, and rose again to 40.1°C after 4 hours. Her blood pressure was 107/62 mmHg, pulse rate 132bpm, respiratory rate 22cpm with oxygen saturation of 94% on room air. Chest examination showed reduced air entry bilaterally and bronchial breath sounds with coarse crepitations. She was given intranasal oxygen at 4L/min. Treatment was started with intravenous (IV) ceftriaxone 2g daily, oral Azithromycin 500 mg stat then 250 mg daily for 3 days, IV Dexamethasone 6 mg daily, SC enoxaparin 40 mg daily for 3 days. Other supplements including oral vitamin C 1g daily for 14 days, oral Zinc 40 mg daily for 14 days, and oral Paracetamol 1g four times daily for 5 days, and intravenous fluids were administered to her.

**Diagnostic assessment:** a nasopharyngeal sample was taken to be tested for SARS-CoV-2 and the patient was nursed in the isolation ward pending results. Blood samples were also collected into blood culture bottle (5ml), anticoagulated tube (3ml) and gel-activator tube (3ml) for blood culture analysis, hematological analysis, and biochemical/immunological analysis, respectively. All samples were transported to the Kumasi Centre for Collaborative Research into Tropical Medicine (KCCR), KNUST, via cold chain (except for blood culture sample, which was transported at room temperature of 24°C) for laboratory analysis. Blood samples were investigated for full blood count, blood culture, renal function test, Lactate Dehydrogenase (LDH), ferritin, and malaria parasites. We also evaluated antibody responses to SARS-CoV-2 by performing IgG and IgM antibody tests on serum samples. SARS-CoV-2 testing was performed [9], starting with genomic extraction of viral agent using Qiagen RNA Minikit (Hilden, Germany), following the manufacturer´s protocol. A real-time polymerase chain reaction (RT-PCR) test was performed on the samples using the Real Star SARS-CoV-2 RT-PCR kit (Altona Diagnostics) using cycling conditions of 55°C for 20 minutes for Reverse Transcription, 95°C for 2 minutes and 15 seconds for denaturation, and 45 cycles of 55°C and 72°C for 45 seconds and 15 seconds, respectively. Patient´s sample tested positive for SARS-CoV-2. The blood culture sample was incubated in Bactec FX40 (BD, France) and incubated at 37°C. The blood culture flagged positive after 24 hours of incubation and was sub-cultured on blood and MacConkey agar. The growth characteristics showed pure non-lactose fermentation on MacConkey agar and non-haemolytic colonies on blood agar. The isolate was subjected to Analytical Profile Index 20NE (Biomerieux, France) testing, and this identified the isolate as *Burkholderia cepacia*. Antimicrobial susceptibility testing showed the isolate was susceptible to ceftriaxone, ceftazidime, chloramphenicol, but resistant to amoxicillin and gentamicin. Other biochemical and haematological investigations were performed using automated chemistry and haematology analyzers. She had low hemoglobin level with neutrophil leukocytosis, elevated lactate dehydrogenase (LDH) and ferritin and normal renal function test. Table 1 gives details of the laboratory results obtained.

**Table 1:** laboratory results for patient

Laboratory test	Results
White blood cell	15.98 x 10^3^ /ul
Haemoglobin	8.7g/dl
Neutrophil	8.17 x 103 /ul
LDH	1016 IU/L
Serum ferritin	3012 ng/ml
Urea	2.3 mmol/l
Creatinine	82 umol/l
Serum SARS-CoV-2 antibodies	
IgG	Positive
IgM	Positive

**Therapeutic intervention:** intravenous paracetamol was administered to help control temperature spikes. On the 4^th^ day of admission, an obstetric ultrasound scan was taken which showed a live intrauterine gestation. On the 5^th^ day, she complained of loss of liquor and an urge to pass stool. A vaginal examination revealed the baby´s head in the introitus. She was immediately positioned for vaginal delivery, as all delivery protocols were observed. However, the baby was stillborn. Nasopharyngeal, oropharyngeal and meconium samples of baby as well as maternal samples were taken for COVID-19 testing and this came back negative. The patient was stable after delivery with vital signs of 102/65mmHg, pulse rate of 132bpm, temperature of 37.2°C, and oxygen saturation of 95% on room air. She was put on intravenous ceftriaxone and discharged after showing good clinical improvement.

**Figure 1:**
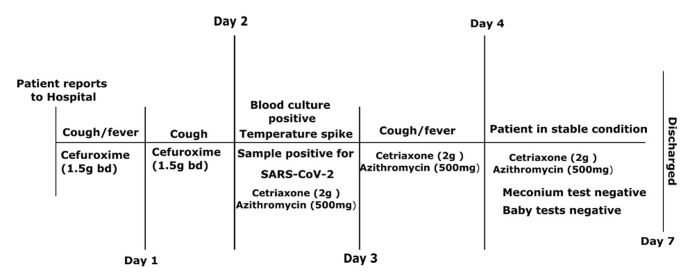
timelines of patient’s disease progression

**Timeline of episode:** Figure 1 describes the history of events.

**Informed consent:** the purpose of the study was explained to the patient, and an informed consent received before samples were collected. The patient was made aware that her medical records will be kept confidential.

## Discussion

The patient in this report presented with characteristic symptoms of COVID-19. She had severe COVID-19 infection, manifested by acute respiratory distress. The pregnancy may have facilitated the disease progression as pregnancy is known to compromise the immune system and therefore, makes pregnant women vulnerable to diseases such as COVID-19 and succumb to its worst outcomes [10]. In our patient, the unsettling temperature patterns as well as the unstable oxygen saturation support the assertion that pregnant women with COVID-19 are more likely to be hospitalized and are at increased risk for Intensive Care Unit (ICU) admission and receipt of mechanical ventilation than non-pregnant women [11]. In a meta-analysis from Mater *et al*. 2020, 37.7% of neonates born to mothers with COVID-19 were preterm, illustrating a slightly higher preterm birth rate compared to the general pregnant population [12]. Infections in utero are known to be a major contributor to preterm delivery. From the same study of mothers with COVID-19, three neonatal deaths were recorded at 34, 31, and 30 weeks of which two were due to multiple organ failure and Disseminated Intravascular Coagulation (DIC), and the third being a perinatal death occurring within 24 hours [12]. Interestingly, in our patient, the baby was born at 32+ weeks, although the exact cause of death could not be ascertained.

At birth, samples of the baby were negative for COVID-19. The placenta was not analyzed histologically for possible acute or chronic inflammatory changes. A case definition of SARS-CoV-2 in pregnant women, fetuses, and neonates classifies neonatal congenital infection as one in which the virus is detected in amniotic fluid before rupturing of membranes or in blood drawn in early life [13]. In a previous study, samples taken from the baby and placenta for COVID-19 PCR came out positive to show a possible case of vertical transmission of the disease. However, in another study, there was no vertical transmission [14]. From Vivanti *et al*. who reported vertical transmission, the placental bed had a very high viral load than the amniotic fluid and maternal blood. It is known that angiotensin 2 converting enzyme inhibitors (ACE2), which are the receptors for SARS-CoV-2, are highly expressed in the placental tissues [13]. As compared to other studies on COVID-19 in pregnancy, this case presents a slightly different picture in that although the patient was confirmed to have severe COVID-19, blood culture which was done at the height of the infection isolated *Burkholderia cepacia*, indicating concurrent sepsis from a different microorganism. *Burkholderia cepacia* infection with high mortality has been reported among patients with cystic fibrosis who have received lung transplantation [15]. The first case of *Burkholderia cepacia* in a patient with COVID-19 has been reported in the United States [7] but none from Africa, except this report. Whilst the first case was a female in her fifties with underlying medical conditions like hypertension, diabetes and hypothyroidism, our patient was a relatively younger African woman with no chronic illness. However, the severity of both patients´ clinical condition was undoubtedly exacerbated by the presence of the bacteria. This seems to explain why our patient continued to have swinging temperature, and the patient in the previous report remained ventilator dependent even after testing negative for COVID-19.

## Conclusion

This report elucidates the deleterious outcome of COVID-19 and *Burkholderia cepacia* co-infection in pregnancy. Clinicians should perform blood cultures in COVID-19 patients presenting with sepsis in order to isolate any potential bacteria and institute appropriate therapy.
